# Supplementary material for: Identification of photoperiod-induced specific miRNAs in the adrenal glands of Sunite sheep (Ovis aries)
Source: Front Vet Sci. 2022 Jul 22;9:888207. doi: 10.3389/fvets.2022.888207 (PMC9354845; doi:10.3389/fvets.2022.888207)
Supplement: Supplementary file 1 [file Data_Sheet_1.ZIP › Supplementary file/miRDeep2 file.zip!/miRDeep2 file/Know_Structure/SPLP42A3/oar-mir-10a.pdf]

miRBase precursor : oar-mir-10a
Total read count : 88772
oar-miR-10a read count : 88215
remaining reads : 557

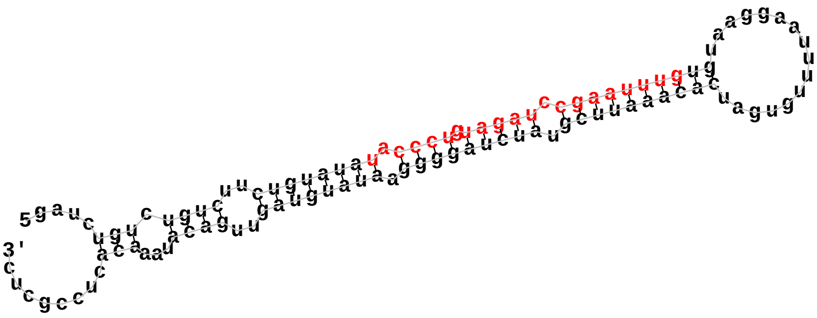

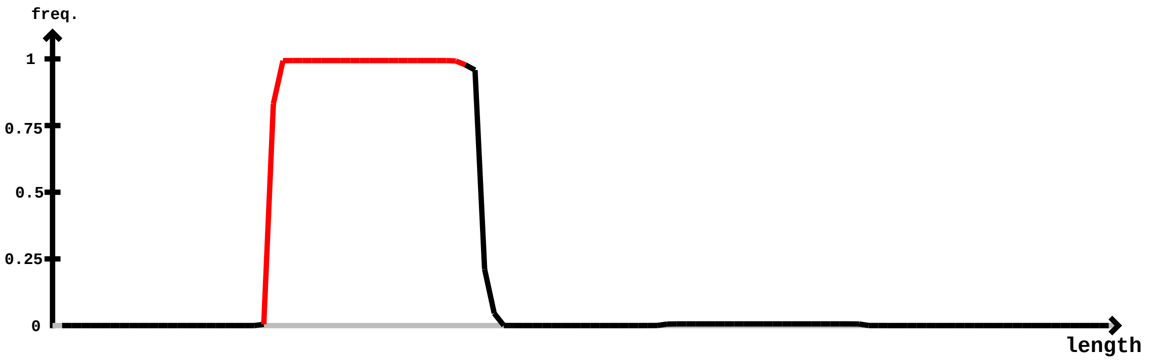


```
                                        oar-miR-10a
5'- gaucugucugucuucuguauauacccuguagauccgaauuuguguaaggaauuuugugaucacaaauucguaucuaggggaauauguaguugacauaaacacuccgcuc   -3'    exp
    ....(((.((((..((((((((.((((.(((((.((((((((((................)))))))))).))))))))).))))))))..))))...)))........    reads    mm    sample
    ..ucugucugucuucuguaua........................................................................................    1        0     seq
    ....................auacccuguagauccgaauu.....................................................................    2        0     seq
    ....................auacccuguagauccgaauuu....................................................................    34       0     seq
    ....................auacccuguagauccgaauuug...................................................................    52       0     seq
    ....................auacccuguagauccgaauuugu..................................................................    284      0     seq
    ....................auacccuguagauccgaauuugug.................................................................    29       0     seq
    ....................auacccuguagauccgaauuugugu................................................................    4        0     seq
    .....................uacccuguagauccgaau......................................................................    10       0     seq
    .....................uacccuguagauccgaauu.....................................................................    116      0     seq
    .....................uacccuguagauccgaauuu....................................................................    1118     0     seq
    .....................uacccuguagauccgaauuug...................................................................    1434     0     seq
    .....................uacccuguagauccgaauuugu..................................................................    57621    0     seq
    .....................uacccuguagauccgaauuugug.................................................................    10620    0     seq
    .....................uacccuguagauccgaauuugugu................................................................    2489     0     seq
    .....................uacccuguagauccgaauuugugua...............................................................    11       0     seq
    .....................uacccuguagauccgaauuuguguaa..............................................................    2        0     seq
    .....................uacccuguagauccgaauuuguguaag.............................................................    1        0     seq
    ......................acccuguagauccgaau......................................................................    1        0     seq
    ......................acccuguagauccgaauu.....................................................................    9        0     seq
    ......................acccuguagauccgaauuu....................................................................    140      0     seq
    ......................acccuguagauccgaauuug...................................................................    257      0     seq
    ......................acccuguagauccgaauuugu..................................................................    8361     0     seq
    ......................acccuguagauccgaauuugug.................................................................    4028     0     seq
    ......................acccuguagauccgaauuugugu................................................................    1534     0     seq
    ......................acccuguagauccgaauuugugua...............................................................    6        0     seq
    ......................acccuguagauccgaauuuguguaa..............................................................    1        0     seq
    .......................cccuguagauccgaauuug...................................................................    1        0     seq
    .......................cccuguagauccgaauuugu..................................................................    23       0     seq
    .......................cccuguagauccgaauuugug.................................................................    10       0     seq
    .......................cccuguagauccgaauuugugu................................................................    11       0     seq
    ........................ccuguagauccgaauuugu..................................................................    3        0     seq
    ........................ccuguagauccgaauuugugu................................................................    1        0     seq
    ........................ccuguagauccgaauuuguguaa..............................................................    1        0     seq
    .........................cuguagauccgaauuugugu................................................................    2        0     seq
    ...........................................................ucacaaauucguaucuagggga............................    3        0     seq
```

oar-miR-10a

```
gaucugucugucuucuguauauacccuguagauccgaauuuguguaaggaauuuugugaucacaaauucguaucuaggggaauauguaguugacauaaacacuccgcuc
............................................................cacaaauucguaucuaggggaa...........................    1    0    seq
............................................................cacaaauucguaucuaggggaau..........................    1    0    seq
.............................................................acaaauucguaucuaggggaa...........................    4    0    seq
.............................................................acaaauucguaucuaggggaau..........................    14   0    seq
.............................................................acaaauucguaucuaggggaaua.........................    1    0    seq
..............................................................caaauucguaucuagggga............................    5    0    seq
..............................................................caaauucguaucuaggggaa...........................    16   0    seq
..............................................................caaauucguaucuaggggaau..........................    440  0    seq
..............................................................caaauucguaucuaggggaaua.........................    9    0    seq
..............................................................caaauucguaucuaggggaauau........................    3    0    seq
...............................................................aaauucguaucuaggggaau..........................    50   0    seq
...............................................................aaauucguaucuaggggaaua.........................    3    0    seq
...............................................................aaauucguaucuaggggaauau........................    4    0    seq
................................................................aauucguaucuaggggaauau........................    1    0    seq
```
